# Influence of Gender in Advanced Heart Failure Therapies and Outcome Following Transplantation

**DOI:** 10.3389/fcvm.2021.630113

**Published:** 2021-02-25

**Authors:** María Dolores García-Cosío, Francisco González-Vilchez, Raquel López-Vilella, Eduardo Barge-Caballero, Manuel Gómez Bueno, Manuel Martínez-Selles, Jose María Arizón, Diego Rangel Sousa, José González-Costello, Sonia Mirabet, Félix Pérez-Villa, Beatriz Díaz Molina, Gregorio Rábago, Ana Portolés Ocampo, Luis de la Fuente Galán, Iris Garrido, Juan F. Delgado

**Affiliations:** ^1^Servicio de Cardiología, Hospital 12 de Octubre Madrid, Instituto de Investigación Sanitaria Hospital 12 de Octubre (imas12), Madrid, Spain; ^2^Centro de Investigación Biomédica en Red Cardiovascular (CIBERCV), Madrid, Spain; ^3^Servicio de Cardiología, Hospital Universitario Marqués de Valdecilla, Santander, Spain; ^4^Servicio de Cardiología, Hospital Universitari i Politecnic La Fe, Valencia, Spain; ^5^Servicio de Cardiología, Complejo Hospitalario Universitario de A Coruña, A Coruña, Spain; ^6^Servicio de Cardiología, Hospital Universitario Puerta de Hierro Majadahonda, Madrid, Spain; ^7^Servicio de Cardiología, Hospital General Universitario Gregorio Marañón, Universidad Europea, Universidad Complutense, Madrid, Spain; ^8^Servicio de Cardiología, Hospital Universitario Reina Sofía, Cordoba, Spain; ^9^Servicio de Cardiología, Hospital Universitario Virgen Del Rocío, Seville, Spain; ^10^Servicio de Cardiología, Hospital Universitari De Bellvitge, Hospitalet de Llobregat, Spain; ^11^Servicio de Cardiología, Hospital Santa Creu i Sant Pau, Barcelona, Spain; ^12^Servicio de Cardiología, Hospital Clínic i Provincial, Barcelona, Spain; ^13^Servicio de Cardiología, Hospital Universitario Central De Asturias, Oviedo, Spain; ^14^Servicio de Cirugía Cardiaca, Clínica Universidad De Navarra, Navarra, Spain; ^15^Servicio de Cardiología, Hospital Universitario Miguel Servet, Zaragoza, Spain; ^16^Servicio de Cardiología, Hospital Clínico Universitario De Valladolid, Valladolid, Spain; ^17^Servicio de Cardiología, Hospital Universitario Virgen De La Arrixaca, Murcia, Spain; ^18^Departamento de Medicina, Universidad Complutense de Madrid, Madrid, Spain

**Keywords:** gender, female, heart transplantation, outcome, women, advanced heart failure, ventricular assist device

## Abstract

Biological differences between males and females change the course of different diseases and affect therapeutic measures' responses. Heart failure is not an exception to these differences. Women account for a minority of patients on the waiting list for heart transplantation or other advanced heart failure therapies. The reason for this under-representation is unknown. Men have a worse cardiovascular risk profile and suffer more often from ischemic heart disease. Conversely, transplanted women are younger and more frequently have non-ischemic cardiac disorders. Women's poorer survival on the waiting list for heart transplantation has been previously described, but this trend has been corrected in recent years. The use of ventricular assist devices in women is progressively increasing, with comparable results than in men. The indication rate for a heart transplant in women (number of women on the waiting list for millions of habitants) has remained unchanged over the past 25 years. Long-term results of heart transplants are equal for both men and women. We have analyzed the data of a national registry of heart transplant patients to look for possible future directions for a more in-depth study of sex differences in this area. We have analyzed 1-year outcomes of heart transplant recipients. We found similar results in men and women and no sex-related interactions with any of the factors related to survival or differences in death causes between men and women. We should keep trying to approach sex differences in prospective studies to confirm if they deserve a different approach, which is not supported by current evidence.

## Introduction

There is a growing interest in sex-related differences in several clinical scenarios. Men and women differ in body composition and physiology; they present differences in pharmacokinetics and pharmacodynamics; and they may also respond differently to cardiovascular drugs. Women are underrepresented in most clinical trials, and real-life data have shown that they are less often treated with evidence-based therapies and experience adverse drug reactions more often (1). The reason for these differences between men and women is beyond the scope of the present study. Still, a better knowledge of these sex-related differences may be helpful to improve patient care.

Most heart failure (HF) patients are female. Women have a different clinical profile than men (2); they develop end-stage HF at an older age, have a higher prevalence of HF with preserved ejection fraction and a lower prevalence of ischemic heart disease (IHD) (3–5). HF prognosis seems to be better in women with a lower rate of premature death than men (4). Moreover, in HF with reduced ejection fraction, women seem to have a better response to treatment, with a more favorable reverse remodeling regardless of the cause and severity of the left ventricle systolic dysfunction (5). In the field of advanced HF, the underrepresentation of women among heart transplant (HT) or ventricular assist devices (VAD) recipients has been attributed to selection and referral bias and potentially poorer outcomes for these therapies. However, whether the described better outcomes in women with HF may also explain this under-representation in advanced heart failure stages has not been explored.

The majority of the studies in the field of heart transplantation (HT) are focused on donor-recipient mismatch (6–8). However, sex-related differences in patients on the waiting list for an HT or ventricular assist device and long term survival after an HT have been addressed recently. We aim to review those topics and look for sex-related differences in 1-year outcomes after an HT in an extensive nationwide registry to elucidate possible gaps that may need further investigation in the future.

## Materials and Methods

### Data Source

The Spanish Heart Transplant Registry is a prospective database promoted by the Heart Failure Working Group of the Spanish Society of Cardiology, containing detailed clinical information about all HT procedures performed in our country from 1984 to the present. The registry is updated yearly with data supplied by all transplant centers in the country (9). The Ethics Committees of all participating centers have approved the Spanish Heart Transplantation Registry for investigational purposes.

For the present study, we included all patients aged ≥18 years who underwent an HT in Spain from January 1, 2005 to December 31, 2019. Vital status at the end of follow-up and cause of death (when applicable) was known for all participants. The cause of death was locally adjudicated in each participating center. We excluded recipients of a second HT and multiorgan recipients.

### Missing Data

Missing data (Supplementary Table 1) were handled by multiple imputations using the wholly conditional specification method, generating 10 imputed datasets using all applicable adjustment variables and the outcome variable as predictors. The average of the 10 imputed data sets was used for analysis. For imputation, categorical and continuous variables were modeled using logistic regression and linear regression, respectively.

### Statistical Analysis

Quantitative variables were summarized as median (interquartile range), and the Mann-Whitney *U*-test assessed between-sex differences. Categorical variables were summarized as percentages, with Chi-squared or Fisher's exact tests, as appropriate, for between-sex comparisons.

The primary outcome was 1-year all-cause mortality or re-transplantation. The associations between baseline population characteristics and outcome were fitted by the use of Cox proportional hazards regression. Multivariable adjustment included the recipient's sex and those variables with a significance level <0.10 in the univariable analysis. To further explore possible differences between men and women, additional multivariable models were considered to include the interaction between the recipient sex and each variable that reached statistical significance in the final multivariable analysis.

All statistical tests were 2-sided, and a *p*-value <0.05 was considered significant. All analyses were performed using the SPSS 25.0 (SPSS Inc., Chicago, IL).

## Results

A total of 3,616 HT procedures were performed in 16 HT centers during the study period. We identified 869 female recipients (24%). Sex-stratified baseline characteristics of the study population are shown in Table 1.

**Table 1:** Characteristics of the study cohort stratified by gender.

	**Female**	**Male**	**P-value**
	**(*n* = 869)**	**(*n* = 2,747)**	
**Recipient**			
Age (years)	54.0 (43.0, 61.0)	57.0 (49.0, 63.0)	<0.001
Etiology (%)			<0.001
Dilated	38.7	36.4	
Ischemic	22.8	45.0	
Others	38.6	18.6	
Predicted heart mass (*g*)	126.8 (118.1, 138.2)	176.0 (164.5, 189.2)	<0.001
Body mass index (Kg/m^2^)	24.0 (21.3, 27.6)	25.5 (23.4, 28.2)	<0.001
Diabetes (%)	12.1	23.3	<0.001
Hypertension (%)	23.2	38.9	<0.001
COPD (%)	6.1	12.2	<0.001
Peripheral vascular disease (%)	2.6	7.8	<0.001
Pretransplant malignancy (%)	8.5	4.1	<0.001
GFR (ml/min/1.73 m^2^)	71.1 (51.4, 95.0)	71.3 (52.8, 94.5)	0.64
GFR <45 mL/min/1.73 m^2^	17.6	15.0	0.08
CMV serology positive	82.3	80.0	0.15
Bilirubin >2 mg/dL	15.3	18.9	0.02
Pulmonary vascular resistance (WU)	2.0 (1.3, 2.9)	2.0 (1.3, 2.8)	0.42
Pre-transplant cardiac surgery (%)	25.3	29.2	0.03
Pre-transplant infection (%)	10.4	14.8	0.001
Pre-transplant mechanical ventilation (%)	15.5	14.4	0.44
Pre-transplant circulatory support (%)			0.002
None	71.1	65.6	
IABP	11.1	13.0	
ECMO	8.1	7.3	
VAD	9.7	14.1	
Recipient location (%)			0.08
Home	56.0	52.5	
Hospital ward	11.4	10.7	
Intensive care unit	32.6	36.8	
**Surgical procedure**			
Urgent transplant (%)	33.8	37.9	0.03
Cold ischemic time (min)	210.0 (153.3, 240.0)	205.0 (155.0, 245.0)	0.94
Surgical technique (bicaval) (%)	61.0	64.3	0.08
Transplant era			0.34
2005–2009	31.4	33.7	
2010–2014	30.1	30.3	
2015–2019	38.4	36.0	
**Donor**			
Age (years)	45.0 (34.0, 53.0)	44.0 (32.0, 52.0)	0.11
Gender (female)	57.3	28.7	<0.001
Predicted heart mass (g)	139.3 (129.0, 152.0)	184.0 (169.5, 199.0)	<0.001
Body mass index (Kg/m^2^)	24.4 (22.5, 26.7)	26.0 (24.0, 28.4)	<0.001
CMV serology positive	74.4	72.3	0.25
Cause of death (%)			0.02
Trauma	24.2	29.0	
CVD	49.3	46.7	
Other	26.6	24.3	
**Donor/recipient interaction**			
Donor/recipient gender mismatch (%)	42.7	28.7	<0.001
Donor/recipient predicted heart mass ratio	1.09 (1.00, 1.12)	1.03 (0.96, 1.13)	<0.001
Donor/recipient CMV serology mismatch (%)	34.9	36.2	0.81
Donor/recipient BMI ratio	1.00 (0.89, 1.16)	1.01 (0.91, 1.14)	0.31

*COPD, Chronic Obstructive Pulmonary Disease; GFR, Glomerular Filtration Rate; CMV, cytomegalovirus; WU, wood units; IABP, Intraaortic balloon pump; ECMO, extracorporeal membrane oxygenator; VAD, ventricular assist device; CVD, cerebrovascular disease*.

Women were significantly younger, and had a lower body mass index and predicted heart mass than men. They also presented with history of neoplastic disease more often.

Men had a poorer cardiovascular risk profile assessed as a higher prevalence of hypertension and diabetes, and triple the prevalence of peripheral artery disease. They also had two times the prevalence of chronic obstructive pulmonary disease. Men had undergone previous cardiac surgery more frequently than women.

HT indication was mainly due to IHD in men. Conversely, in women, HT's leading cause was dilated cardiomyopathy (DCM), followed by other etiologies (valvular heart disease, congenital heart disease, hypertrophic cardiomyopathy, restrictive cardiomyopathy, and myocarditis).

Urgent HT and mechanical circulatory support (VAD and intra-aortic balloon pump) were more frequent in men. Abnormal bilirubin levels and active infection at the moment of HT were also more frequent in men.

Although women received grafts from female donors who had a lower body mass index and predicted heart mass more often, donor/recipient sex mismatch was more frequent. Consequently, donor/recipient predicted heart mass ratio was higher.

Median follow-up was 1.01 years (interquartile range 0.71–1.01). Results of the univariate and multivariate Cox regression analysis are summarized in Table 2. Variables related to impaired survival included recipient age, higher body mass index, diabetes mellitus, bilirubin of >2 mg/dl, pre-HT infection, previous cardiac surgery, need for mechanical ventilation at the moment of HT, mechanical circulatory support at the time of HT, recipient location in the Intensive Care Unit, urgent transplant, cold ischemic time, female donor and donor/recipient sex mismatch, and donor/recipient body mass index ratio. Higher glomerular filtration rate, bicaval surgical technique, and HT in the recent period (2015–2019) were related to a better outcome. After multivariate analysis, body mass index and diabetes of the recipient lost statistical significance as did any type of mechanical circulatory support at the time of HT, recipient location at the time of HT, urgent status, female donor, and donor-recipient body mass index.

**Table 2:** Uni- and multivariate Cox regression analysis of 1-y survival.

	**Univariate**			**Multivariate**		
	**HR**	**CI (95%)**	***P*-value**	**HR**	**CI (95%)**	***P*-value**
**Recipient**						
Female gender	1.11	0.94–1.30	0.21	1.15	0.97–1.36	0.10
Age (years)	1.01	1.01–1.02	<0.001	1.01	1.00–1.02	0.005
Predicted heart mass (g)	1.00	1.00–1.00	0.59			
Body mass index (Kg/m^2^)	1.02	1.00–1.04	0.02	1.01	0.97–1.03	0.68
Etiology						
Dilated	1					
Ischemic	1.11	0.94–1.30	0.22			
Other	1.16	0.97–1.40	0.11			
Diabetes	1.18	1.00–1.40	0.05	1.11	0.93–1.33	0.23
Hypertension	1.13	0.98–1.32	0.10			
COPD	1.09	0.87–1.36	0.48			
PVD	1.20	0.92–1.56	0.18			
GFR (mL/min/1.73 m^2^)	0.99	0.99–0.99	<0.001	0.99	0.99–1.00	<0.001
CMV serology positive	1.10	0.92–1.33	0.29			
Bilirubin >2 mg/dL	1.49	1.25–1.78	<0.001	1.35	1.13–1.61	0.001
PVR (Wood U.)	1.05	1.00–1.11	0.08	1.02	0.97–1.08	0.42
Pre-transplant infection	1.60	1.34–1.92	<0.001	1.34	1.10–1.63	0.004
Pre-transplant cardiac surgery	1.32	1.14–1.53	<0.001	1.22	1.04–1.43	0.02
Mechanical ventilation	2.00	1.70–2.37	<0.001	1.64	1.32–2.05	<0.001
Pre-transplant circulatory support						
None	1			1		
IABP	1.45	1.18–1.77	<0.001	1.10	0.88–1.37	0.41
ECMO	1.69	1.34–2.15	<0.001	1.20	0.89–1.63	0.23
VAD	1.39	1.14–1.71	0.001	1.21	0.94–1.57	0.15
Recipient location^a^						
Home	1					
Hospital ward	1.15	0.90–1.46	0.27			
Intensive care unit	1.57	1.35–1.82	<0.001			
Pre-transplant malignancy (%)	1.19	0.89–1.61	0.24			
**Surgical procedure**						
Urgent transplant	1.51	1.31–1.74	<0.001			
Cold ischemic time (min)	1.00	1.00–1.00	<0.001	1.00	1.00–1.00	<0.001
Surgical technique (bicaval) (%)	0.78	0.68–0.90	0.001	0.85	0.73–0.99	0.03
Transplant era						
2005–2009	1			1		
2010–2014	0.90	0.76–1.07	0.24	0.91	0.77–1.09	0.30
2015–2019	0.72	0.61–0.86	<0.001	0.70	0.58–0.85	<0.001
**Donor**						
Age (years)	1.00	1.00–1.01	0.22			
Gender female^a^	1.20	1.07–1.34	0.001			
Predicted heart mass (g)	1.00	1.00–1.00	0.33			
Body mass index (Kg/m^2^)	0.99	0.98–1.01	0.50			
Cause of death						
Trauma	1					
CVD	0.95	0.81–1.13	0.56			
Other	1.00	0.83–1.21	0.99			
CMV serology positive	0.88	0.75–1.04	0.12			
**Donor/recipient interaction**						
Donor/recipient gender mismatch	1.16	1.00–1.34	0.049	1.22	1.05–1.42	0.01
Recipient/Donor CMV mismatch						
No	1					
Donor (−)/recipient (+)	1.14	0.96–1.37	0.14			
Donor (+)/recipient (−)	0.91	0.73–1.15	0.43			
Donor/recipient predicted heart mass ratio	0.92	0.55–1.54	0.75			
Donor/recipient body mass index ratio	0.65	0.45–0.95	0.02	0.80	0.50–1.28	0.35

*HR, Hazard ratio; CI, Confidence interval; COPD, Chronic obstructive pulmonary disease; CVD, cerebrovascular disease; ECMO, extracorporeal membrane oxygenator; GFR, glomerular filtration rate; IABP, intra-aortic balloon pump; PVD, peripheral vascular disease; PVR, pulmonary vascular resistance; VAD, ventricular assist device*.

a *Urgent transplant, recipient location, and donor sex were not included in the multivariate model due to collinearity with Pre-transplant Circulatory Support and recipient and donor sex*.

In the final model, variables independently related to reduced survival were recipient age, history of previous cardiac surgery, bilirubin of >2 mg/dl, pre-HT infection, need for mechanical ventilation at the moment of HT, cold ischemic time, and donor/recipient sex mismatch. Higher glomerular filtration rate, bicaval surgical technique, and HT in the recent period (2015–2019) were independently associated with a better prognosis.

**Figure 1:**
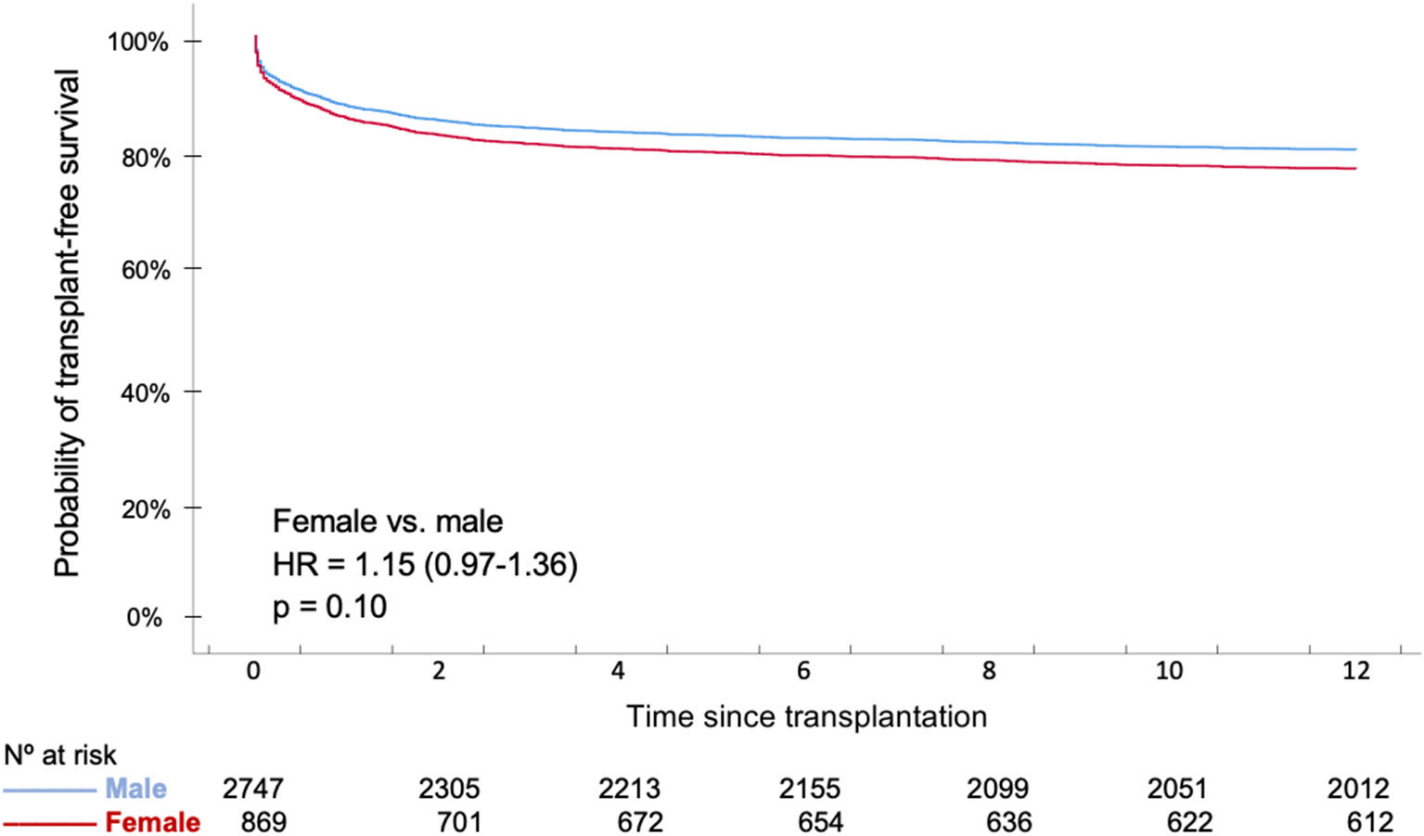
Adjusted 1-year survival curves according to recipient sex.

**Figure 2:**
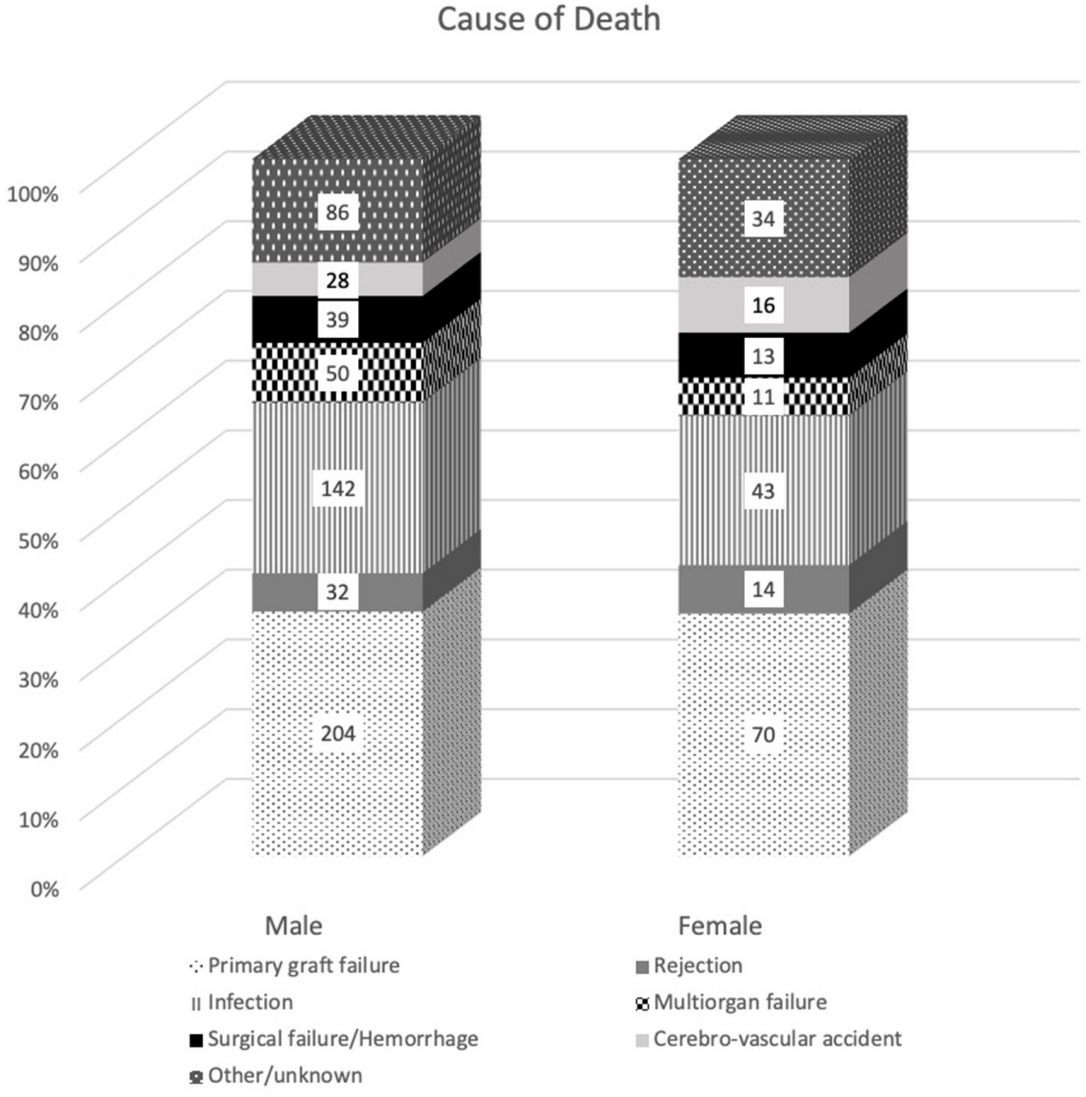
Causes of death segregated by sex. Data are expressed as count (percentage in the y-axis). Chi-square = 6.075; *P* = 0.41.

Women and men had a similar 1-year survival (women 76.4 vs. 78.6% men *p* = 0.34) by adjusted Kaplan-Meier analysis (Figure 1). We did not find any differences in the cause of death between men and women (Figure 2).

We did not find any interaction between sex and variables independently related to survival in the multivariate analysis (Table 3).

**Table 3:** Analysis of interactions between recipient gender and significant variables in multivariate analysis.

**Variable**	**Recipient female gender**			**Interaction**		
				recipient sex * variable		
	**HR**	**CI (95%)**	***P*-value**	**HR**	**CI (95%)**	***P*-value**
Recipient age	0.83	0.36–1.90	0.66	1.01	0.99–1.02	0.46
GFR (mL/min/1.73 m^2^)	1.01	0.64–1.60	0.95	1.00	0.99–1.01	0.64
Bilirubin >2 mg/dL	1.08	0.89–1.30	0.43	1.21	0.81–1.79	0.35
Pre-transplant infection	1.07	0.89–1.28	0.49	1.34	0.89–2.04	0.17
Pre-transplant cardiac surgery	1.08	0.88–1.31	0.47	1.14	0.81–1.61	0.46
Mechanical ventilation	1.11	0.92–1.34	0.30	1.06	0.73–1.54	0.76
Bicaval surgical technique	1.22	0.95–1.55	0.12	0.87	0.62–1.20	0.39
Cold ischemic time	0.81	0.46–1.44	0.47	1.00	1.00–1.00	0.24
Transplant era	1.04	0.79–1.37	0.76			
2010–2014				1.20	0.81–1.77	0.37
2015–2019				1.05	0.70–1.56	0.81
Donor/Recipient gender mismatch	1.11	0.89–1.38	0.35	1.03	0.74–1.43	0.88

*GFR, glomerular filtration rate*.

## Discussion

Advanced heart failure affects 1–10% of the overall HF population and implies a severe decline in patients' quality of life and survival. The Heart Failure Association of the European Society of Cardiology has recently updated diagnostic criteria. It focuses on patient referral to advanced HF centers and a proper transition of patients to palliative care (10). Although there are interesting HF registries to gather information about HF patients' clinical parameters and characteristics and their therapies, the advanced heart failure population is somehow challenging to study and scarcely described in the literature. Gender differences in HF patients have been previously described, but their clinical implications remain unclear. A better knowledge of the sex-related differences appears as a potential field of improvement in the diagnosis, treatment, and likely prognosis of HF patients. The more significant publications addressing sex differences in advanced HF patients (waiting-list, HT, and VAD) are summarized in Table 4.

**Table 4:** Summary of recent publications addressing gender differences in patients on the waiting for a heart transplant, receiving a long term ventricular assist device, or heart transplant recipients.

**Publication**	**Population**	**Period**	**Analysis**	**Conclusions**
Hsich et al. (11)	28852 PWL 24% women (SRTR)	2000–2010	Propensity Score Long term survival	Women higher risk in urgent status Similar results in intermediate status in men and women Men higher risk in elective status
Hsich et al. (12)	33069 PWL 25% women (SRTR)	2004–2015	3 year survival Random survival forest	Higher risk in urgent and intermediate status in women, similar in recent period Higher risk in elective status in men Multiple interactions between sex in different status
Magnussen et al. (13)	966 VAD (75% BTT) 15% women (EUROMACS)	2011–2014	1–2-year survival	Similar HT rates Women worse survival Women sicker at implant Women higher major bleeding, arrhythmias, and RV failure
DeFilippis et al. (14)	13305 VAD 20.8% women (UNOS)	2008–2018	Propensity Score 1-2-year survival	Increase in VAD use among decade (lower in women) Women sicker at implant, similar complications Women lower HT rate and survival
Ahmed et al. (15)	3511 VAD 23.3% women (NIS)	2009–2014	Propensity Score In hospital survival	Similar survival Similar complications VAD in females have doubled lately
Hickey et al. (16)	345 HT 28% women NEW HEART study	2011–2015	1-year survival	Similar survival Women younger Women more rejection episodes and hospitalizations
Moayedi et al. (17)	34198 HT 23.7% women (ISHLTR)	2004–2014	Propensity Score Adjusted IMPACT / DRI Long-term survival	Similar survival Lowest survival in undersized donors Women higher mortality in regular sized donors
García-Cosío et al. (18)	6740 HT 20.6% women (SHTR)	1997–2017	Temporal trends Transplant rate pmh Long term survival	Similar survival Similar HT pmh in women among 25 years (lower in men) Women died due to rejection and primary graft failure Men died due to malignancies
Current series	3616 HT 24% women (SHTR)	2005–2019	1-year survival	Similar survival

*PWL, Patients on the waiting list; SRTR, USA Scientific Registry of Transplant Recipients; VAD, long term ventricular assist device; BTT, bridge to transplantation; EUROMACS, European Registry for Patients with Mechanical Circulatory Support; HT, Heart transplant; RV, right ventricle; UNOS, United Network for Organ Sharing; NIS, USA National Inpatient Database; ISHLTR, International Society of Heart and Lung Transplantation Registry; IMPACT, Index for Mortality Prediction After Cardiac Transplantation score; DRI, Donor Risk Index; SHTR, Spanish Heart Transplant Registry; Pmh, per million habitants*.

Previous studies with a small sample of patients showed a worse survival rate for women on the waiting list for HT (19). Several analyses of the Scientific Registry of Transplant Recipients of the United States of America have assessed the same topic. Hsich et al. in 2014 analyzed sex differences in patients listed for HT in 10 years (2000–2010) stratified by severity of illness (1A, high risk; 1B, intermediate risk; and 2, low-risk ambulatory patients) and adjusted by baseline characteristics. Women accounted for 25% of the study population, and they had a higher mortality rate than men in urgent status (1A) but a lower mortality rate than men in an elective ambulatory setting (status 2). No differences were observed in the intermediate-risk status 1B (11). Women were younger and had a non-ischemic cardiomyopathy more often, and men had a worse cardiovascular risk profile, and IHD was the leading cause for HF. The same authors tried to confirm these sex differences in a more recent period (2004–2015) and attempted to identify factors associated with waiting-list mortality and transplantation timing. Although similar differences in mortality were observed between 2004 and 2008 (higher mortality in woman in status 1A and 1B and lower in status 2), in the most recent years, some of them were solved, and women had a similar survival in urgent status (1A) and elective status (2). They also identified many sex interactions for death and HT that varied with prioritization on the waiting list that should be addressed as a new field to understand these differences between men and women (12). Improvements in the risk of death or deterioration in women waiting for HT have also been observed in other studies (20).

There is also available information about sex-related differences in VAD therapy. As expected, baseline characteristics and underlying comorbidities and etiologies differed between men and women as it has been described for studies of patients on the HT waiting list. An analysis of the European Registry for Patients with Mechanical Circulatory Support showed that only 15% of patients receiving a VAD were women. HT rates were similar for men and women. However, women were at a more advanced stage at the moment of implantation, presented a higher rate of significant bleeding, arrhythmias, and right ventricular failure, and had a worse prognosis than men (13). Two studies in the United States of America also showed a lower use of VAD in women, although slightly higher than in the European Registry (21–23%). This higher percentage of women undergoing a VAD implantation might be explained by the inclusion of patients listed in a more recent time-lapse. Increasing use of VAD therapy in women throughout the observation period is described. Both American registries represent conflicting results on survival. The former is focused on in-hospital survival and showed similar survival for men and women (15). The latter evaluated more extended follow-up periods and described lower HT rates and a lower survival in women (14). In both registries, women presented with a more severe HF, but a similar adverse event rate. Those differences in outcomes may reflect different follow-up times and, thus, different rates of adverse events in men and women after perioperative period.

Several factors might play a role in sex-related differences in HT outcomes: the higher frequency of anti-HLA antibodies detection in women, differences in predicted heart mass as a critical factor in donor-recipient matching, and variability in clinical presentation men and women (21, 22). The New Heart study was the first one to address this topic. They found a similar survival for HT in women, who represented 28% of the analyzed population. Conversely, women were younger and developed graft rejection and needed hospitalization more often than men (16). An analysis of the International Society of Heart and Lung Transplantation Registry, in which 23.7% of included patients were women, also showed similar survival rates after adjusting by recipient and donor risk scores but suggested a higher mortality rate to women who received a graft of a regular-sized donor (17). Our previous work analyzing the Spanish Heart Transplant Registry results over the last 25 years showed a similar survival and similar HT rate in women per million habitants. Causes of death differed between men, mainly due to neoplastic diseases, and women, mainly due to primary graft failure and rejection (18). All the described studies show a comparable pattern of baseline characteristics and underlying heart disease.

Our study aims to describe sex-related differences in 1-year outcomes after an HT in a contemporary cohort. Given that previous studies showed sex-related differences in higher-risk recipients, we sought to analyze 1-year HT results as they may be affected considerably by perioperative factors like the patient's clinical situation on the waiting list or the etiology of HF.

We did not find differences in the recipient's location (outpatient or hospitalized) at the time of HT. The need for circulatory support at the moment of HT was more frequent in men (mainly VAD), but it was not associated with different outcomes. VADs were used in a low percentage of HT candidates in our cohort. Given that VAD therapy may have different results in men and women, we cannot extrapolate our results to other populations with a higher VAD use. Urgent HT was more frequent in men, but it was not associated with higher mortality after multivariate analysis. We cannot determine whether this difference is influenced by a higher delisting rate of women due to clinical deterioration before HT or different use of therapies that determine urgent status in our country (i.e., intra-aortic balloon pump until 2015, extracorporeal membrane oxygenator, or VAD).

To conclude, despite sex-related differences in the clinical profile and the donor-recipient matching, 1-year outcomes are comparable. We did not find differences in the cause of death, and we did not find any interactions between sex and factors significantly associated with differences in survival.

## Limitations

Our analysis of 1-year outcomes after HT has some limitations that must be acknowledged. The main limitation is the lack of information about patients included on the waiting list for HT since the patient's follow-up begins at HT. Thus, we do not have any data about those patients who are included on the waiting list and are delisted or died before HT. Another limitation is the low rate of VAD implantation in our cohort that prevents us from extrapolating these results to other populations. Furthermore, the retrospective nature of a registry analysis also constitutes a significant limitation.

## Conclusion

Women are under-represented in the waiting list for an HT or a VAD. Although clinical profile and HF etiology differ between men and women, overall survival and complications are similar. It is desirable to study sex-related differences to understand if we should adjust clinical protocols in advanced HF patients by sex.

## Data Availability Statement

The raw data supporting the conclusions of this article will be made available by the authors, without undue reservation.

## Ethics Statement

Ethical review and approval was not required for the study on human participants in accordance with the local legislation and institutional requirements. The patients/participants provided their written informed consent to participate in this study.

## Author Contributions

MG-C, JD, and FG-V have contributed to the conception and design of the work. MG-C and FG-V have contributed to the analysis and interpretation of the work's data and drafting. BM, RL-V, EB-C, MG, MM-S, JM, DR, JG-C, SM, FP-V, GR, AP, LdlF, and IG have contributed in revising the article critically. All authors contributed to the article and approved the submitted version.

## Conflict of Interest

The authors declare that the research was conducted in the absence of any commercial or financial relationships that could be construed as a potential conflict of interest.

## Abbreviations

DCM: Dilated cardiomyopathy
HF: Heart Failure
HT: Heart transplant
IHD: ischemic heart disease
VAD: Ventricular assist device.
