# Supplementary material for: Influence of Gender in Advanced Heart Failure Therapies and Outcome Following Transplantation
Source: Front Cardiovasc Med. 2021 Feb 25;8:630113. doi: 10.3389/fcvm.2021.630113 (PMC7946818; doi:10.3389/fcvm.2021.630113)
Supplement: Supplementary file 1 [file Table_1.DOCX]

Table 1S: Missing data

| Variable | N | % |
| --- | --- | --- |
| **Recipient** |  |  |
| Age (years)^a^ | 0 | 0.0 |
| Gender (%) | 0 | 0.0 |
| Etiology (%) | 0 | 0.0 |
| Predicted heart mass (g)^a^ | 32 | 0.9 |
| Body mass index (Kg/m^2^) | 32 | 0.9 |
| Diabetes (%) | 46 | 1.3 |
| Hypertension (%) | 138 | 3.8 |
| COPD (%) | 197 | 5.4 |
| Peripheral vascular disease (%) | 67 | 1.9 |
| GFR (ml/min/1.73 m^2^) ^a^ | 153 | 4.2 |
| CMV serology positive | 84 | 2.3 |
| Bilirubin > 2 mg/dL | 293 | 8.1 |
| Pulmonary vascular resistance (WU) ^a^ | 610 | 16.9 |
| Pre-transplant cardiac surgery (%) | 59 | 1.6 |
| Pre-transplant infection (%) | 37 | 1.0 |
| Pre-transplant mechanical ventilation (%) | 61 | 1.7 |
| Pre-transplant circulatory support (%) | 21 | 0.6 |
| Recipient location (%) | 76 | 2.1 |
| Pretransplant malignancy (%) | 149 | 4.1 |
| **Surgical procedure** |  |  |
| Urgent transplant (%) | 0 | 0.0 |
| Cold ischemic time (min) ^a^ | 59 | 1.6 |
| Surgical technique (bicaval) (%) | 65 | 1.8 |
| Transplant era (%) | 0 | 0.0 |
| **Donor** |  |  |
| Age (years) ^a^ | 36 | 0.1 |
| Gender (female) | 27 | 0.7 |
| Predicted heart mass (g)^a^ | 107 | 3.0 |
| Body mass index (Kg/m^2^) | 99 | 2.7 |
| CMV serology positive | 340 | 9.4 |
| Cause of death (%) | 0 | 0.0 |
| **Donor/recipient interaction** |  |  |
| Donor/recipient gender mismatch (%) | 27 | 0.7 |
| Donor/recipient predicted heart mass ratio | 136 | 3.2 |
| Donor/recipient CMV serology mismatch (%) | 385 | 10.6 |
| Donor/recipient BMI ratio | 108 | 3.0 |

COPD: Chronic Obstructive Pulmonary Disease; GFR: Glomerular Filtration Rate; CMV: cytomegalovirus; WU: wood units; BMI: Body mass index.
